# Evaluation of CAG repeat length in the androgen receptor gene and polycystic ovary syndrome risk in Iranian women: A case-control study

**DOI:** 10.18502/ijrm.v20i3.10711

**Published:** 2022-04-21

**Authors:** Hamideh Arasteh, Fatemeh Araste, Mohammad Hasan Sheikhha, Ph.D., Seyyed Mehdi Kalantar, Ehsan Farashahi Yazd, Hamid Reza Ashrafzadeh, Nasrin Ghasemi

**Affiliations:** ^1^Department of Medical Genetics, International Campus, Shahid Sadoughi University of Medical Sciences, Yazd, Iran.; ^2^Department of Medicinal Biotechnology, School of Medicine, Shahid Sadoughi University of Medical Sciences, Yazd, Iran.; ^3^Abortion Research Center, Yazd Reproductive Sciences Institute, Shahid Sadoughi University of Medical Sciences, Yazd, Iran.

**Keywords:** *Androgen receptor*, * (CAG)_n_ repeats*, * Polycystic ovary syndrome.*

## Abstract

**Background:**

Polycystic ovary syndrome (PCOS) is a heterogeneous disorder, which affects about 15-20% of women of reproductive age. The most important etiopathogenesis factor in its incidence is hyperandrogenism; over 70 candidate genes are known to be associated with this syndrome, such as the androgen receptor (*AR*) gene which encodes a steroid receptor and is located on the Xq11-12 chromosome. The N-terminus of exon 1 of *AR* contains a polymorphic trinucleotide repeat (CAG)_n_ region that encodes glutamine tract. There are some studies showing that shorter *AR* CAG repeats are significantly related to enhanced *AR* sensitivity.

**Objective:**

This study investigated the frequency of the polymorphic expansion of the trinucleotide CAG repeats of *AR* in PCOS.

**Materials and Methods:**

160 Iranian women aged 17-40 yr participated in this case-control study: 80 women as PCOS patients and 80 women as healthy controls according to the Rotterdam criteria. Other similar phenotype factors such as hyperandrogenism were not considered as PCOS. The frequency of polymorphic expansion of CAG trinucleotide repeats in PCOS patients was compared with the frequency in non-PCOS controls in using two primer sets for nested polymerase chain reaction. The polymerase chain reaction products were visualized on polyacrylamide gel and then were confirmed by a sequencing process.

**Results:**

The results did not show a significant correlation between the frequency of CAG repeats in *AR* and PCOS incidence.

**Conclusion:**

In contrast to some previous reports, the present data showed that the CAG length in PCOS cases did not significantly differ from that of controls. So, the *AR* (CAG)_n_ does not appear to be a major factor for PCOS in Iranian women.

## 1. Introduction

Polycystic ovary syndrome (PCOS) is one of the most common endocrine disorders in young women. Polycystic refers to an excess of several hundred microcysts which are detected in an ultrasound on one or both of the ovaries (1). The syndrome commonly presents with hyperandrogenism and patients with PCOS suffer from irregular menstruation, unintentional weight gain and/or increased accumulation of fat around the abdominal area (central adiposity), androgenetic alopecia, excessive virilization and hirsutism (2). Moreover, the risk of developing cardiovascular diseases, infertility, diabetes mellitus type 2 and endometrial hyperplasia in PCOS patients is increased (3, 4). The relationship between PCOS and breast cancer is also a controversial subject (3, 4). Although the pathogenesis of the syndrome is largely unclear, there is evidence that both environmental and genetic factors influence the incidence of PCOS: studies on susceptible individuals have shown that healthy-weight women with PCOS present with higher insulin resistance (5). Furthermore, the chance of PCOS incidence in patients' sisters is about 50% (6). It has been shown that in the early stages of primate development, poor conditions such as prenatal androgenization, impaired nutrition, and an imbalanced steroidal environment can lead to the development of PCOS phenotype (7). By 2021, 70 candidate genes related to PCOS had been introduced; some of them are involved in biosynthesis and metabolism of steroids, cholesterol and lipids (8). Most of the PCOS-influencing genes are related to androgen biosynthesis and metabolism such as androgen receptor (*AR*), gonadotropins, and sex hormones (9). The *AR* gene includes eight exons and seven introns and is located on the X chromosome (Xq11-q12 region). The first exon of *AR* is associated with a polymorphic CAG repeated region that encodes polyglutamine residues (10). Previously, it has been reported that CAG repeat expansions to more than 40 triplets in *AR* can be correlated with spinobulbar muscular atrophy (11). Similarly, some reports have shown that a shorter repeat of CAG tract elevates the sensitivity of *AR*, which subsequently leads to some hyperandrogenism-related disorders such as androgen-dependent prostate cancer, benign prostatic hyperplasia and juvenile rheumatoid arthritis (12). Correlations have also been found between CAG repeats in *AR *and infertility in males, increased risk of colon cancer and Alzheimer's disease in men (13). In the case of PCOS, despite some studies indicating an association between abnormal CAG repeats, some others have suggested that the relationship is not reasonable as it has been confirmed that even within the normal morphology, the range of CAG polymorphism is between 11 to 35 repeats (12, 14-16).

The aim of this study was to evaluate the relationship between CAG repeats in *AR* in Iranian women and PCOS occurrence as a hyperandrogenic disorder.

## 2. Materials and Methods

### Study population

This case-control study was performed in Yazd Reproductive Sciences Institute, Yazd, Iran, during 2016-2018. 160 women aged between 17-40 yr were recruited into the PCOS (n = 80) and control (n = 80) groups. PCOS was diagnosed according to the Rotterdam criteria by presenting at least two of the following three clinical symptoms: oligomenorrhea or amenorrhea; clinical and/or laboratorial hyperandrogenism; and/or polycystic ovaries diagnosed by ultrasonography, as accepted by the National Institutes of Health (17). Women with other hyperandrogenism factors with similar phenotypes as PCOS such as Cushing's syndrome were excluded. Data on age, height, weight, menstrual characteristics, serum testosterone level, hirsutism and luteinizing hormone / follicle-stimulating hormone ratio were recorded for the two groups.

### Sampling

Vein blood samples (5 cc) were collected from each woman in both groups, were combined with EDTA as an anticoagulant agent and then stored at -20 C. The genomic DNA was extracted from each blood sample using a DNA purification kit (Roje Technologies, Yazd, Iran). Using the previously reported primers (18), a nested polymerase chain reaction (PCR) was used to isolate the CAG repeat fragments in exon 1 of *AR*. In the first round of PCR, 17 cycles using the 5'-GTGCGCGAAGTGATCCAGAA-3' and 5'-TCTGGGACGCAACCTCTCTC-3' sequences as forward and reverse primers were performed (94 C for one min, 55 C for one min, and 72 C for 1.5 min). The inside PCR was performed by the 5'-ACTCTCTTCACAG-CCGAAGAAGGC-3' and 5'ATCAGGTGCGGTGAAGTCGCTTTCC-3' primers. The final products were analyzed by electrophoresis on an agarose gel (2% agarose). For analysis of the exact nucleotide length, PCR products were confirmed using 5% denaturing polyacrylamide gels. Finally, to determine the exact CAG repeat number, the resulting fragments were sequenced after the second round of PCR.

### Ethical considerations

All women were willing to participate and signed a written consent form and the study proposal was approved by the Ethics Committee of Shahid Sadoughi University of Medical Sciences, Yazd, Iran (Code: 183154).

### Statistical analysis

Statistical analyses were performed using the Statistical Package for the Social Sciences software version 20.0 (SPSS Inc., Chicago, IL, USA). The association between different alleles and the risk of PCOS was examined by the Chi-square test and odds ratios. The sample size was calculated based on the prevalence of CAG repeats in both groups with a confidence level of 95%, and accuracy of 5%, using the following formula:

N = (z1-α)2×(1-p)/d2

The CAG repeats in *AR* were considered as the independent variable and the other parameters, including serum testosterone level, hirsutism, luteinizing hormone / follicle-stimulating hormone ratio and irregular menstruation, were analyzed as dependent variables.

## 3. Results

**Figure 1:**
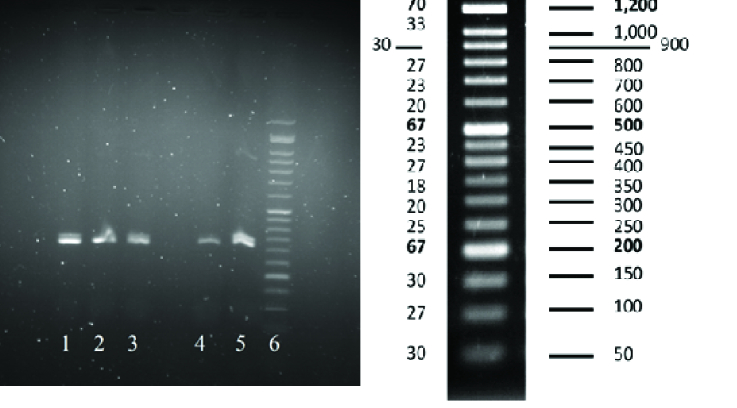
Agarose gel electrophoresis of the first round of PCR. A 324 bp band confirms the product. Lane 1-3: Samples from individuals in the PCOS group, Lane 4, 5: Samples from individuals in the control group, Lane 6: 50 bp DNA ladder.

Using two sets of primers in two separate reactions, the specificity of the PCR products was increased for the later sequencing process. PCR results were visualized on gel electrophoresis. The first round of PCR product was run on 2% agarose gel; as shown in figure 1, the product was placed between the 300 and 350 lanes of the ladder, representing the 324bp band and confirming the accuracy of the PCR process.

**Figure 2:**
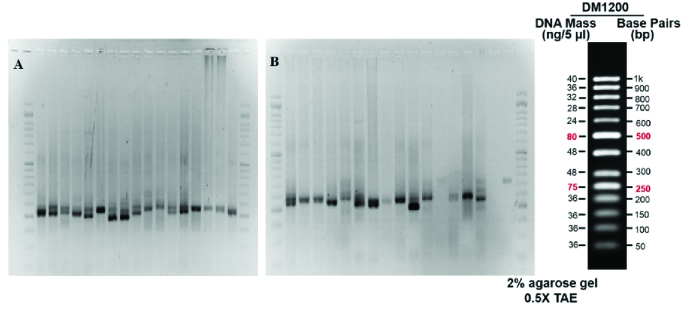
Agarose gel electrophoresis of the second round of PCR of (A) Samples from individuals in the PCOS group, (B) Samples from individuals in the control group. The first and last wells indicate the 50bp DNA ladder. A 230bp band of samples confirms the nested PCR process. A double band in some samples represents the heterozygosity.

**Figure 3:**
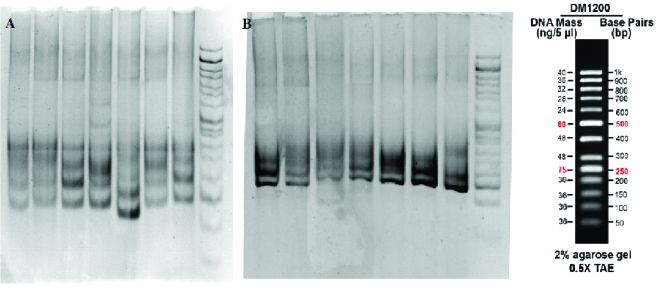
Polyacrylamide gel electrophoresis of the second round of PCR of (A) Samples from individuals in the PCOS group, (B) Samples from individuals in the control group.

At the second round of amplification, the PCR products of the first round were used for multiplication of a 230bp fragment which was confirmed on agarose and polyacrylamide gels. As shown in figure 2, the products were placed between the 200 and 250 bands of the ladder on 2% agarose gel, indicating the desired process of amplification. The CAG triplet length of the nested-PCR products was also analyzed on polyacrylamide gel electrophoresis containing 5% acrylamide. The resulting band around 230bp reconfirmed the PCR process (Figure 3).

**Figure 4:**
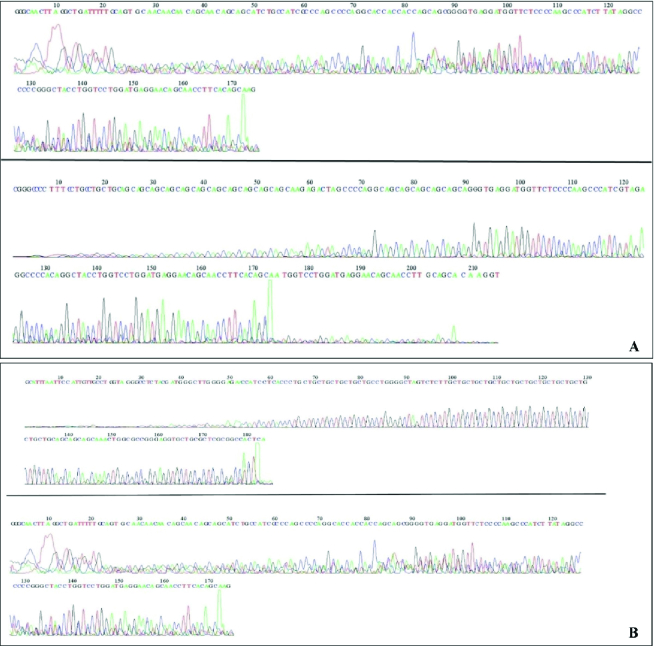
An example of the sequencing results of samples from (A) Control and (B) PCOS individuals.

In order to determine the exact length of the CAG repeats, products of the second round of the nested-PCR process were sequenced; examples of the sequencing results are shown in figure 4.

Based on the sequencing process, three alleles with different ranges of CAG repeat lengths were identified. As shown in table I, the largest CAG length of *AR* was 9-21 repeats and the smallest length of CAG repeats was 23-30 in both the control and PCOS groups. There was no significant difference in the length of CAG repeats between the control and PCOS groups (p = 0.52). The heterozygote and homozygote frequencies in both groups were evaluated by Chi-square test (Table I); the heterozygote population was larger than the homozygotes with no statistically significant difference between the number of heterozygotes and homozygotes. There were 13 and nine individuals with a homozygote natural allele (22 repeats) in the PCOS and control groups, respectively, and 14 and 15 women with heterozygote mutant alleles in the PCOS and control groups, respectively (Table I). There was no significant difference between heterozygotes and homozygotes (p = 0.61) in the PCOS and control groups, and also no significant difference between natural and mutant allele homozygotes (p = 0.44). The PCOS group had 22 CAG repeats and the control group had less than 22 CAG repeats (p = 0.96, odds ratio = 1.015, and confidence interval = 0.51-2.00), so there was no significant association between the CAG repeat frequency and incidence of PCOS.

**Table 1:** Three alleles based on CAG repeats, their heterozygotes and homozygotes, and homozygote natural and mutant alleles frequencies


**Groups**		**PCOS**	**Control**
**CAG repeats**			
	**9-21**	51 (63.75)	46 (57.50)
	**22**	27 (33.75)	24 (30.00)
	**23-30**	2 (2.50)	10 (12.50)
**Heterozygote**		53 (66.25)	56 (70.00)
**Homozygote**		27 (33.75)	24 (30.00)
**Homozygote natural allele**		13 (48.10)	9 (37.50)
**Homozygote mutant alleles**		14 (51.90)	15 (62.50)
Data presented as n (%), PCOS: Polycystic ovary syndrome			

## 4. Discussion

In the present study we investigated whether the CAG repeat length polymorphisms in the *AR *gene were associated with PCOS. To our knowledge, this is the first study comparing Iranian women with PCOS and healthy controls which evaluates the effect of CAG repeat polymorphisms on PCOS. Although the role of genetic factors in PCOS is strongly supported, there is no known direct genetic link to PCOS.

Some studies have shown a correlation between different polymorphisms of *AR* and androgen function in these patients (16, 19). There is some evidence showing that PCOS leads to conditions related to increased levels of androgens, such as oligo-anovulation, infertility, multifollicular ovary, hirsutism, inhibition of oocyte maturation, acne and androgenic alopecia (10, 20).

Furthermore, increased androgen levels during fetal and childhood stages can cause abdominal obesity, amenorrhea and insulin resistance (20). It has been shown that increased androgen levels is a key factor in the pathology of PCOS (19). Several studies have indicated that special polymorphisms in exon 1 of *AR* strongly affect the activity of the *AR*, for example, an increased number of CAG repeats leads to lower *AR* activity (21).

Our results showed that there was no significant relationship between the frequency of CAG repeats in this exon and the incidence of PCOS. These results are in agreement with previous studies reported about Greek, Croatian and Finnish women (16, 22), and other studies similarly reporting that *AR *is not a major determinant of PCOS (23). The number of heterozygotes in our study was larger than the homozygotes; however, this difference was not statistically significant. In this area, a study on Romanian populations showed that in homozygotes, the CAG repeat length in PCOS women was significantly shorter in comparison to healthy women (24). In the same way, a study on American women showed lower frequencies of CAG repeats in homozygotic PCOS women (15). It is of note that in some studies the results have been the opposite; in their study on Australian women, Hicky et al. reported a higher frequency of short (CAG)_n_ alleles in PCOS patients in comparison with the controls (25). Some contradictory results were also obtained in evaluations of the direct correlation between increased androgen symptoms such as puberty and acne, and CAG repeat frequencies in PCOS patients. The data showed no correlation between the CAG frequencies and such clinical symptoms (14, 22, 26), which was in contrast to other studies that have presented a direct and meaningful relationship between lower CAG repeats and puberty and/or acne (27).

When considering these paradoxical and varied results it should be noted that *AR* is located on a highly conserved region of the X chromosome; therefore, given the variation in epigenetic factors including X inactivation (XCI), the phenotype of individuals cannot always be predicted from their genotype (27). In some situations XCI occurs non-randomly; it has been shown that this phenomenon can have a significant impact on the incidence of PCOS (15). Animal and human studies have shown that certain environmental conditions such as prenatal exposure to androgen excess can affect fetal programming and cause alterations in postnatal growth and metabolism, leading to a higher prevalence of PCOS (28). These findings indicate that fetal exposure to environmental stressors and abnormal conditions influence the DNA methylation pattern. It is hypothesized that factors affecting epigenetic changes such as DNA methylation and the XCI process have potential effects on *AR* and the incidence of PCOS (29). Varied results have been observed in the XCI pattern of PCOS: some studies have reported an association between CAG repeats and XCI in women with PCOS (23), while another study reported that there was no difference found in the XCI pattern of PCOS patients (15).

There are several possible reasons why the aforementioned studies have produced inconsistent and conflicting results. First, this may be due to the genetic variation of different nations. The normal range of CAG repeats in African-American and Asian people are longer than in European people (30). Second, the existing studies considered different sample sizes, ranging from 80 women in our study, to 300 women in the Shah et al. report (15). Third, results might vary across studies because of epigenetic and XCI effects on *AR *expression. Cell regulatory mechanisms and transcription factors affecting *AR *expression should be evaluated (10). In the functioning of *AR*, as a dynamic heterocomplex, a large number of co-activators and co-repressors have been identified as critical mediators for tissue-specific androgen effects (15).

## 5. Conclusion

In conclusion, we examined the association between CAG repeats of exon 1 of *AR* and PCOS in the Iranian population as a prognosis marker. The results of this study rejected the hypothesis that CAG repeat polymorphisms in *AR *can be a determinant of PCOS.

##  Conflict of Interest

The authors declare that there is no conflict of interest.
